# A 45-year-old female patient with Sheehan’s syndrome presenting with imminent adrenal crisis: a case report

**DOI:** 10.1186/s13256-021-02827-0

**Published:** 2021-05-08

**Authors:** Abere Genetu, Yibeltal Anemen, Sinshaw Abay, Simachew Anemen Bante, Kebadnew Mulatu Mihrete

**Affiliations:** 1 Department of Internal Medicine, College of Medicine and Health Sciences, Bahir Dar University, Bahir Dar, Ethiopia; 2 Department of Midwifery, College of Medicine and Health Sciences, Bahir Dar University, Bahir Dar, Ethiopia; 3 Department of Epidemiology and Biostatistics, School of Public Health, College of Medicine and Health Sciences, Bahir Dar University, Bahir Dar, Ethiopia

**Keywords:** Case report, Panhypopituitarism, Sheehan’s syndrome, Postpartum hemorrhage, Secondary adrenal insufficiency, Adrenal crisis, Central hypothyroidism

## Abstract

**Background:**

Sheehan's syndrome is hypopituitarism due to pituitary gland necrosis resulting from hemorrhagic shock during pregnancy. It is a rare complication with varied manifestations and a considerable delay in diagnosis.

**Case presentation:**

We describe the case of a 45-year-old Ethiopian woman who presented with generalized fatigue for 18 years which progressed to anorexia, nausea, vomiting, diarrhea, and abdominal pain of 6 years' duration, for which she was treated symptomatically throughout these years. Complete clinical evaluation, endocrine studies, and pituitary magnetic resonance scan revealed hypopituitarism secondary to Sheehan’s syndrome. She had significant improvement noted following the commencement of hormone replacement therapy.

**Conclusion:**

Previous case reports describe patients being diagnosed after one or more complications from long-term panhypopituitarism. The present case illustrates that undiagnosed Sheehan's syndrome is associated with long-term morbidity, and we want to emphasize that a high index of suspicion is crucial for the early diagnosis of the syndrome in routine clinical visits in order to prevent complications arising with delayed diagnosis. Awareness among clinicians is also essential so that such cases are not overlooked, especially in developing nations, where home delivery is still common and obstetric care is limited.

## Background

Sheehan's syndrome is hypopituitarism due to postpartum ischemic necrosis of the pituitary gland. It was first described in 1937 by Sheehan [1]. It is rare complication which occurs in 1 out of every 100,000 births globally and is the most common cause of hypopituitarism in low- or middle-income countries [2, 3]. It is reported that Sheehan’s syndrome accounts for 0.5% of all known cases of hypopituitarism in females [4]. The disease is deemed “rare” in industrialized nations, but in developing nations, due to a lack of access to sophisticated medical procedures, skilled professionals, and medical resources, which contributes to a higher prevalence of postpartum hemorrhage and subsequent Sheehan’s syndrome, it is said to occur in 5 out of every 100,000 births [5, 6]. The prevalence is much higher in developing countries, with a prevalence as high as 3.1% in a state in India where more than half of the affected individuals had home deliveries [7]. The underlying process leading to Sheehan’s syndrome is the infarction of the physiologically enlarged anterior pituitary lobe (due to hyperplasia of prolactin-secreting cells as a result of elevated estrogen secretion) and secondary to the compression of the blood vessels supplying the gland by the enlarged gland itself or due to grossly decreased blood supply during intrapartum or postpartum events. Although other etiologies including vasospasm, autoimmunity, small sella size, and disseminated intravascular coagulation may also play a role in the development of Sheehan’s syndrome, none has been conclusively proven [8]. Sheehan’s syndrome can present during the postpartum period or several months or years following delivery. A study in France showed a delay of 9 ± 9.7 years in the diagnosis of Sheehan's syndrome [9], and a longer delay of 20.37 ± 8.34 years was noted in developing countries [10]. Women with Sheehan’s syndrome have varying degrees of hypopituitarism, ranging from panhypopituitarism to only selective pituitary deficiencies [11, 12]. The most common initial symptoms of Sheehan’s syndrome are agalactia and/or amenorrhea. Uncommonly, it can present as an emergency condition with circulatory collapse, severe hyponatremia, diabetes insipidus, hypoglycemia, congestive cardiac failure, or psychosis [13]. In some cases, the diagnosis is not made until years later, when features of hypopituitarism, such as secondary hypothyroidism or secondary adrenal insufficiency, become evident in a woman who had a postpartum hemorrhage [14].

## Case presentation

**Fig. 1:**
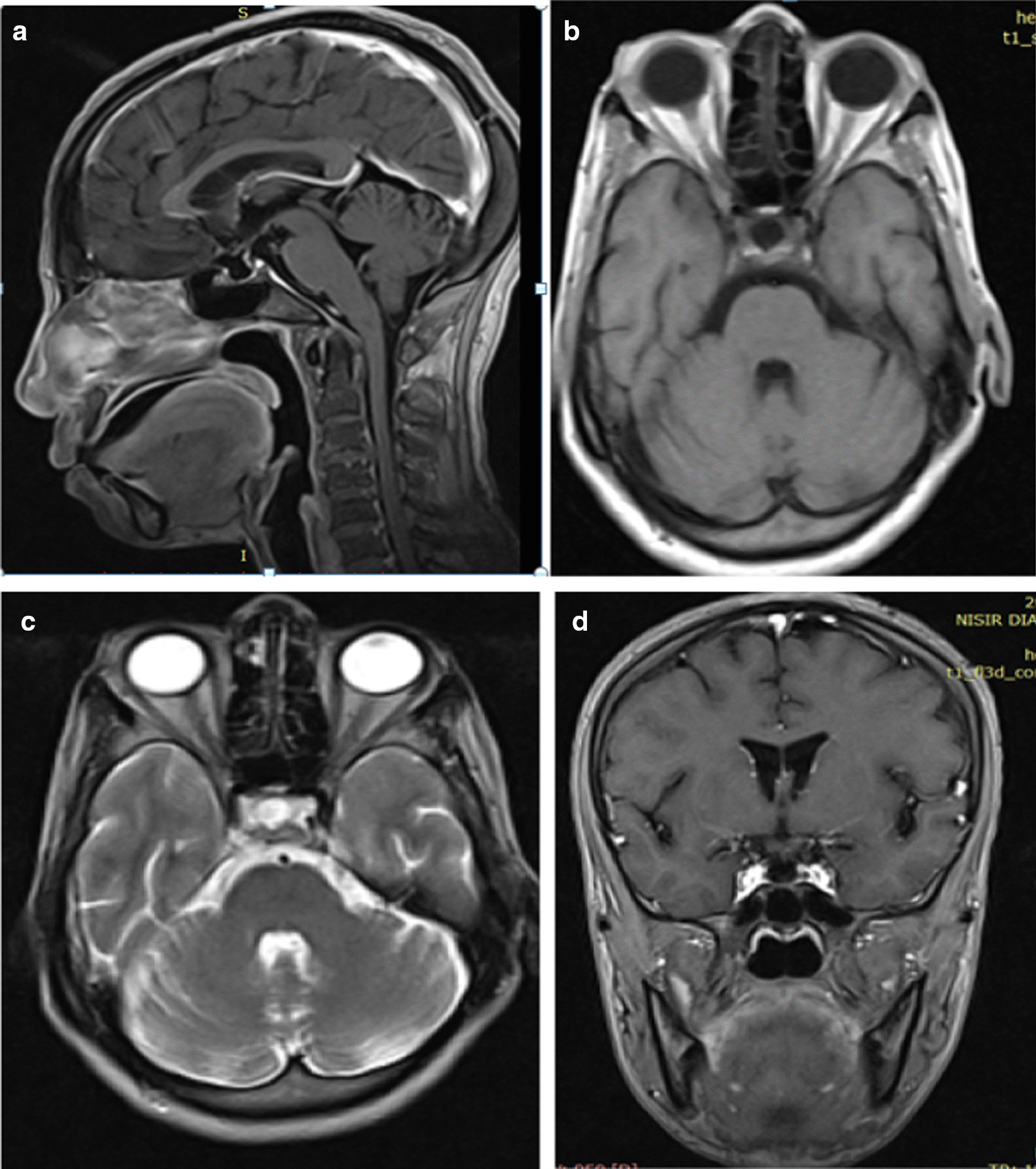
Brain magnetic resonance imaging shows an empty sella. **a** T1-weighted post contrast, **b** T1-weighted axial, **c** T2-weighted axial, **d** T1-weighted coronal

A 45-year-old woman from Achefer, near Bahir Dar, northwestern Ethiopia, presented to the emergency department of our hospital on February 18, 2020, with a complaint of generalized fatigue for 18 years and worsening abdominal pain, vomiting, and diarrhea of 1 month duration. The history dated back 18 years to the birth of her seventh child in home delivery, following which she experienced excessive vaginal bleeding due to delayed expulsion of the placenta. She had lost consciousness following the vaginal bleeding and had been treated locally by traditional healers and survived. After the delivery, she had failed to lactate and remained amenorrheic and could not conceive, but had no any gynecological evaluation. She also complained of progressive generalized fatigue and decreased work capacity, preventing her from doing her routine activities. She had experienced repeated abdominal pain, nausea, vomiting, and diarrhea as well for the past 6 years and had repeatedly visited a nearby clinic and was managed for dyspepsia and gastroenteritis but with no lasting improvement. In addition, she had associated weight loss, anorexia, dizziness, cold intolerance, myalgia, arthralgia, and progressive loss of axillary and pubic hair. These symptoms had worsened over the past month, and she had developed generalized weakness and extreme fatigue even at rest and experienced excessive sleepiness. She also had mild to moderate intermittent headache, but otherwise no blurring of vision or diplopia She had no history of head trauma, surgery, or irradiation, and no history of polyuria or polydipsia on physical examination, and she was conscious but sleepy. Her blood pressure was 90/60 mmHg, pulse rate 86 beats per minute, respiratory rate 24 breaths per minute, and temperature of 36.9 °C. She had mild epigastric and periumbilical tenderness. She had dry, coarse skin and sparse axillary and pubic hair. Examination of other systems was unremarkable. Laboratory findings showed the following: white blood cell count 2590 cells/μL, hematocrit 26.9%, mean corpuscular volume (MCV) 81.3 fl, creatinine 1.2 mg/dL, normal liver function tests, sodium 126 mEq/L, potassium 2.52 mEq/L (repeat 4.2 mEq/L), random blood sugar 113 mg/dL, total cholesterol 152 mg/dL, triglyceride 86 mg/dL, and low-density lipoprotein (LDL) 161 mg/dL. Brain magnetic resonance imaging (MRI) revealed an empty sella turcica, as shown in Fig. 1. As the clinical manifestations related to the patient’s previous obstetric history suggested Sheehan’s syndrome, relevant available hormone studies were performed (Table 1). After all these tests were performed, she was diagnosed with Sheehan’s syndrome presenting with imminent adrenal crisis.

**Table 1:** Laboratory findings of the patient’s hormone profile at presentation

Plasma hormone	Patient’s values	Reference range
Thyroid-stimulating hormone (μIU/mL)	2.26	0.35–4.94
Plasma cortisol at 8 a.m. (μg/dL)	0.9	3.7–19.4
Free thyroxine (ng/dL)	0.35	0.61–1.12
Prolactin (mg/dL)	2.45	5.18–26.53
Luteinizing hormone (mIU/mL)	1.4	5.16–61.99 postmenopausal women
Follicle-stimulating hormone (mIU/mL)	4.2	26.72-133.41 postmenopausal females

The patient was rehydrated with one bag of normal saline (NS), and started on prednisolone 2.5 mg in the evening/5 mg in the morning and subsequently levothyroxine 75 μg/day to be titrated based on clinical response. She was strictly instructed on the nature of her illness and was told to take these medications for the rest of her life. She was referred to an endocrine clinic and discharged. On subsequent clinical assessment, abdominal pain, nausea, vomiting, and diarrhea had subsided; her appetite had returned and she had started to gain weight.

## Discussion

The diagnosis of Sheehan’s syndrome is determined by the patient’s history and physical examination, and confirmed by laboratory tests. Hemorrhagic shock during pregnancy is a key leading point in diagnosis. Failure to lactate is often a common initial complaint in patients with Sheehan syndrome [15]. Many of them also report amenorrhea after delivery [16]. The diagnosis of Sheehan’s syndrome is not made until several years later in certain cases, when the features of hypopituitarism become apparent in a woman who had postpartum bleeding [17]. A woman with Sheehan’s syndrome with undiagnosed hypopituitarism might be apparently asymptomatic until her body is exposed to stressful situations like surgery or infection many years after her delivery, and then she presents with adrenal crisis [18]. Sheehan’s syndrome presents with varied symptoms depending on the specific anterior pituitary hormone deficiencies. Prolactin deficiency can cause lactation failure. Gonadotropin deficiency will often cause amenorrhea or genital hair loss. Corticotrophin deficiency can result in generalized fatigue, weakness, hypoglycemia, or dizziness. Growth hormone deficiency causes fatigue, decreased quality of life, and weight loss. Symptoms of central hypothyroidism are clinically similar to primary hypothyroidism, but patients with central hypothyroidism have low triiodothyronine and thyroxine levels, with normal or even inappropriately low thyroid-stimulating hormone levels. Diagnosis of panhypopituitarism is straightforward, but partial deficiencies are often difficult to determine [19]. Sheehan's syndrome can be acute or chronic [20]. Acute cases present with failure to lactate or amenorrhea. Our patient could not breastfeed following her pregnancy due to lactation failure, and became amenorrheic, indicating an acute presentation. In the previously mentioned study in France, the mean diagnostic delay was 2.52 ± 3 months for patients with agalactia and 8.3 ± 8 years for patients with amenorrhea [9]. Moreover, our patient also developed signs and symptoms of chronic Sheehan’s syndrome, which include secondary adrenal insufficiency such as asthenia, anorexia, and weight loss progressing to dizziness, nausea, vomiting, and abdominal pain, for which she had repeatedly visited health care providers, but the diagnosis was missed. The French study found that the delay in diagnosis in patients presenting with hypothyroidism was 8.1 ± 8.5 years and in those presenting with acute adrenal insufficiency was 10.6 ± 9.4 years [9]. In patients who present with acute disease progressing to chronic conditions, the diagnosis could have been made at several stages. In our patient, the first clue to her diagnosis was her lactational failure and amenorrhea, and the next clue was the manifestation of symptoms of adrenal insufficiency in subtle ways with fatigue and anorexia which progressed to dizziness, nausea, vomiting, and abdominal pain, all of which were missed as findings in making a diagnosis. This can be attributed to a lack of awareness, especially given that patients with panhypopituitarism present with varied nonspecific symptoms, coupled with a lack of a thorough history and physical examination required to diagnose a rare disease.

Laboratory tests can reveal many other anomalies, including hyponatremia. This is the most common electrolyte imbalance, occurring in 33–69% of cases [21, 22]. There are several possible mechanisms by which hypopituitarism can result in hyponatremia. Hypothyroidism can cause decreased free-water clearance and subsequent hyponatremia. Glucocorticoid deficiency can also cause decreased free-water clearance, independent of vasopressin. Hypopituitarism itself can stimulate vasopressin secretion and can cause severe inappropriate secretion of antidiuretic hormone, which can also cause hyponatremia. The potassium level in these situations is normal, because adrenal production of aldosterone is not dependent on the pituitary. In this case the initial hypokalemia noted could be due to gastrointestinal loss following diarrhea and vomiting. The patient’s sodium level subsequently normalized with commencement of hormone replacement therapy, and potassium was corrected with intravenous potassium chloride (KCL) administration.

Anemia is well recognized as a feature of hypopituitarism. Gokalp *et al*. recently reported hematological abnormalities in 65 patients with Sheehan’s syndrome, 80% of whom presented with anemia, compared with 25% of controls [3]. Many hormonal deficiencies, including hypothyroidism, adrenal insufficiency, and gonadal hormonal deficiency, can explain normochromic anemia in hypopituitarism [23]. Pancytopenia is rarely observed in patients affected with Sheehan’s syndrome, and a literature review reveals the rarity this disorder. Our patient had bicytopenia with mild normochromic normocytic anemia. The possibility of Sheehan’s syndrome was suspected because of her obstetric history, signs and symptoms of chronic adrenal insufficiency, hyponatremia, and baseline hormone levels. MRI study of the pituitary gland may reveal different features depending on the stage of the disease. While early scans are not usually helpful for diagnosis, they demonstrate a nonhemorrhagic enlargement of the pituitary gland, leading to its subsequent involution, and late scans typically show an empty sella. A secondary empty sella is considered a characteristic finding in the classical form of Sheehan’s syndrome [24]. Treatment of young women with hypopituitarism usually includes replacement of hydrocortisone first and then replacement of thyroid-stimulating hormone and estrogen with or without progesterone, depending on whether the woman has a uterus. Hydrocortisone is replaced first because thyroxin therapy can exacerbate glucocorticoid deficiency and theoretically induce an adrenal crisis [16, 25]. The standard dose of hydrocortisone is 20 mg/day for an adult (15 mg every morning and 5 mg every evening). Both thyroxin replacement and gonadotropin replacement are common, and doses are titrated to each individual. Replacement of growth hormone is necessary in children with hypopituitarism but is controversial in adults. Some people with severe growth hormone deficiency derive great benefit from replacement, but standard recommendations are not available [26]. For our patient, we replaced relevant and available hormones considering her age, fertility desire, and affordability.

## Conclusion

Thus, although it is rare, a high index of suspicion for Sheehan’s syndrome by primary care physicians is warranted in patients with an obstetric history of intrapartum or postpartum hemorrhage. Sheehan’s syndrome is associated with increased morbidity and mortality if not diagnosed early. A detailed medical history and physical examination supported by laboratory tests is still the cornerstone of diagnosis, reminding clinicians to keep in mind rarely reported diseases like Sheehan’s syndrome. Increased awareness and timely diagnosis can help patients avoid a poor quality of life that can span several years and can prevent precipitating complications.

## Data Availability

The data sets generated during the study are available from the corresponding author upon request.

## Abbreviations

KCL: Potassium chloride
LDL: Low-density lipoprotein
MCV: Mean corpuscular volume
MRI: Magnetic resonance imaging
NS: Normal saline
